# Evaluation of an integration community project for asylum seekers in Sweden: physical activity adherence and changes in character traits and life satisfaction

**DOI:** 10.1038/s41598-024-72413-z

**Published:** 2024-09-13

**Authors:** Matheus Guerra, Daniel Berglind, Maryam Kazemitabar, Erik Lindskär, Erica Schütz, Casimiro Dias, Danilo Garcia

**Affiliations:** 1 Department of Global Health, Karolinska Institute, Stockholm, Sweden; 2 Promotion of Health and Innovation (PHI) Lab, International Network for Well-Being, Linköping, Sweden; 3 Center for Epidemiology and Community Medicine (CES), Region Stockholm, Stockholm, Sweden; 4 School of Medicine, Yale University, New Haven, USA; 5 Department of Psychology, Linnaeus University, Kalmar, Sweden; 6 Pan American Health Organization (PAHO/WHO), Kingston, Jamaica; 7 Department of Social Studies, University of Stavanger, Stavanger, Norway; 8 Lab for Biopsychosocial Personality Research (BPS-PR), International Network for Well-Being, Linköping, Sweden; 9 Department of Behavioral Sciences and Learning, Linköping University, Linköping, Sweden; 10 Centre for Ethics, Law and Mental Health (CELAM), University of Gothenburg, Gothenburg, Sweden; 11 Department of Psychology, University of Gothenburg, Gothenburg, Sweden

**Keywords:** Psychology, Public health

## Abstract

Asylum seekers’ traumatic experiences in combination with discrimination, social isolation, and exclusion in the host country leads to low adherence from health and integration initiatives. Along with their inability to seek health care and physical inactivity, this situation increases their mental illness and, most importantly, decreases their well-being. In fact, the lack of well-being (e.g., life satisfaction) is a better marker of mortality and morbidity than the presence of mental illness. In this context, one of the major single determinants of well-being is character, a dimension of personality that stands for self-regulation, adaptation, and intentional conscious behavior (i.e., goals and values). Host countries often implement integration initiatives including activities aiming to attenuate mental illness, but only a handful are evaluated and reported, with even fewer addressing character development, increases in life satisfaction, or adherence. Our aim was to evaluate the integration initiative “Health for Everyone—Sport, Culture, and Integration”, a 10-week physical activity community project. Specifically, we investigated changes in life satisfaction and character traits (i.e., Self-Directedness, Cooperativeness, and Self-Transcendence) and if these variables, at baseline, predicted adherence and changes in physiological health (i.e., cardiorespiratory fitness, skeletal muscle mass, body fat mass, and visceral fat). Participants (*n* = 269) answered (pre and post measurements) the Satisfaction with Life Scale, the Short Character Inventory, and undertook physiological tests. In addition, their attendance to the physical activity sessions was registered throughout the project (i.e., adherence). Participants showed no significant increases in Self-Directedness, Cooperativeness, or life satisfaction, but significant decreases in Self-Transcendence. Moreover, higher life satisfaction and lower Self-Transcendence at baseline predicted higher adherence to the activity. However, neither character traits nor life satisfaction predicted changes in physiological health. We argue that low frequency physical activity initiatives may improve this population’s physical health because participants probably have a sedentary life and low levels of physical health due to their asylum conditions (e.g., unemployment, low income, poor housing and social network). Furthermore, physical activity per se may not improve the well-being of asylum seekers. Hence, promoting well-being and character development might require person-centered initiatives focusing on the whole individual in order to fit programmes to the needs and life situation of this population.

## Introduction

Throughout human history, adaptation and resilience have been necessary in order to survive when populations are forced into displacement from their native lands. A variety of causes lead people to involuntary migration from their homelands, such as famine (e.g., Ireland 1845–1852, Sweden 1890–1910, Ethiopia 1983–1985); economic deterioration and the collapse of social systems (Venezuela 1999-present, Somalia 1991-present); ethnic persecution (European Jews in Nazi-occupied Europe 1941–1945, Rohingya people in Myanmar 2016- present); and civil war (El Salvador 1979–1992, Rwanda 1990–1994). In this century, the wars in Afghanistan (2001–2021), Iraq (2013–2017), Syria (2011–present), and Ukraine (2020–present), among others, have caused an increase in asylum seekers in Europe. The civil war in Syria, for example, resulted in what became widely known within Europe as the 2015 European migrant crisis—internationally known as the Syrian refugee crisis. Although this crisis refers to a period of increased migration occurring since 2010, it is often associated with the year 2015, when an influx of approximately 1.3 million people requested asylum and receive legal protection in the European Union^1^. Sweden, for example, had 162,877 individuals applying for asylum (cf. 28,575 between 2000 and 2010) with a majority being Syrian (31.5%), Afghani (25.5%) and Iraqi (13.4%) during this period^2^. Thus, presenting many challenges for the asylum seekers and host countries, some of which are still being discussed in Sweden and the rest of Europe until this very day as integration failures and leading to anti-emigrant narratives^3^. However, to the best of our knowledge, studies being currently published concerning the health status of Middle Eastern refugees in Sweden, use data collected in the period of 2016–2018. Therefore not reflecting the current situation of this population’s mental health. One reason for this gap in the literature is that refugees and asylum seekers who arrived during that time period are now either citizens or have residency permits in Sweden. Another more relevant reason though, is that the influx of refugees and asylum seekers from outside the EU, specially from the Middle East, has been extremely restricted by significant policy changes and tightening restrictions^3,4^.

Regardless of country of origin or reasons to flee, the majority of asylum seekers are exposed to traumatic events such as violence, family separations, homelessness, and other forms of trauma, which frequently result in different forms of mental illness, like post-traumatic stress disorder, depression, and anxiety^5^. Indeed, despite the fact that asylum seekers are a heterogeneous group regarding background and health status, research shows a higher prevalence of mental health illness among asylum seekers compared to host populations^6–12^. In Sweden, previous studies show an increased risk of depression in immigrants compared to native Swedes^13,14^. A population-based survey, for example, with a sample of 1,215 Syrian refugees who were granted permanent residency in Sweden found a prevalence of 40.2% of depression, 31.8% of anxiety and 29.9% of post-traumatic stress disorder^15^. In another Swedish study^16^, a total of 56.0% and 58.4% reported clinically significant symptoms of depression and anxiety, with a prevalence of depressive symptoms being five times higher and anxiety four times higher than the general Swedish population. Even after crossing borders and reaching safety and protection, asylum seekers still face hardships in their host countries. For example, during the peak of the Syrian refugee crisis in 2015, the Swedish Migration Board’s average time to reach a decision for asylum applications was 229 days^2^. In fact, the long wait for the resolution of their asylum application in combination with an increase in unfamiliar day-to-day situations, prolonged bureaucratic processes at different authorities, limited language skills, and racism seem to influence asylum seekers to refrain from pursuing and receiving the mental health care they need, slow down social participation and integration, and diminish their opportunities to be physically active^16^. Altogether, this situation deteriorates their physical, mental, and social health, especially when slow logistic and bureaucratic processes prolong the exposure to all the social risk factors mentioned here^16,17^.

### Health is not only the absence of mental illness: promoting well-being

Although the World Health Organization accentuates that achieving the highest attainable standard of health is a fundamental right of every person regardless of race, religion, political belief, economic or social condition^18^; most studies and initiatives on asylum seekers’ health focus on reducing mental illness. Ergo, asylum seekers’ health needs to be addressed beyond the absence of disease (e.g., not showing symptoms of depression and anxiety) and include the promotion of well-being. Indeed, the lack of well-being is more predictive of subsequent mortality and morbidity than the presence of mental illness^19^. Well-being is a multidimensional concept that includes various aspects of mental and physical health, supporting social relationships, and the ability to cope with stressful situations^20,21^. Well-being can be further understood as a state of happiness and fulfillment combined with good physical and mental health, and good quality of life^22^. One of the most studied forms of well-being is subjective well-being, which’s subjective nature captures individual differences in how people judge objective circumstances depending on individual goals, values, and culture^23^. This makes subjective well-being one of the best proxies for measuring, studying, and understanding human well-being^24^.

Life satisfaction, along positive and negative affect, is one of the major components of subjective well-being^25^. It entails the contentment and attainment of an individual’s self-imposed ideal^26^. Life satisfaction has been used to compare people from different countries and cultures, as well as to indicate a person's overall state of physical and psychological health^27^. Refugees and asylum seekers who report significantly higher levels of life satisfaction score higher in host-country-specific language proficiency, social contacts, and feelings of relatedness^28^. Suggesting that the opportunities in the host country are essential for a positive integration process in relation to the individual’s satisfaction with life (for more research on life satisfaction among refugees see elsewhere^29–31^). Moreover, previous studies in different cultures^32–35^ indicate that individuals with higher life satisfaction are more likely to engage in healthy behaviors, to adopt preventive measures in their lifestyles, have better cardiovascular health, and are more inclined to access recommended preventive health care services and to engage in health-enhancing behaviors (e.g., adherence to treatment). Hence, changes in life satisfaction after participation in integration initiatives might be seen as a good indicator of increases in health and lifestyle changes among asylum seekers and a good indicator of positive integration. Additionally, it can be expected that asylum seekers’ life satisfaction at the beginning of health and integration initiatives should predict attendance to the activities (i.e., adherence), thus, leading to positive outcomes after such projects.

### A personality dimension that promotes well-being and healthy behaviors: character

Many factors promote well-being, including access to medical care, social factors, environmental and physical influences, but also genetics. In this context, personality has emerged as a particular determinant of health-related behaviors, physiological and mental health, and preventive behaviors^36,37^. The reason for this has been argued to be the biopsychosocial nature of personality^38–40^. Importantly, the basic functions of personality are to feel, think, and perceive, and to integrate these functions into purposeful behaviors that flexibly and creatively express our identity under changing conditions^38^, such as forced migration.

The common denominator of different definitions of personality is that they focus on phenomena related to a person’s motivation, learning styles, and mental adaptation. From an evolutionary perspective, personality has been defined as the dynamic organization of the biopsychosocial systems by which a person shapes and adapts in a unique way to a changing internal and external environment^38^. Structurally, personality can be decomposed into temperament, which involves basic emotions, such as, fear, anger, disgust, sadness, and joy; and character or what the person makes of herself intentionally in relation to the self (i.e., Self-Directedness), others (i.e., Cooperativeness), and something beyond the self (i.e., Self-Transcendence), such as, humanity, nature, the universe, or God^39^. Furthermore, while temperament is helpful in predicting the type of mental illness, character is valuable in assessing the absence of mental illness and the presence of well-being^38,41–43^. More recently, longitudinal genetic studies (i.e., genome-wide association studies) in three different samples from three different countries (Germany, Finland, and Korea), using personality measures of temperament and character along with other types of data (e.g., parental reports, sociodemographic), show that temperament traits are associated with genes that are stable throughout life and that character traits are associated with genes that are behind epigenetic processes. In other words, the genes behind character, unlike the genes behind temperament, can be activated by external factors (e.g., interventions, personal choices, etc.) without the need to alter DNA^44–47^.

Indeed, longitudinal studies show that temperament and character traits follow different developmental trajectories—while temperament is relatively stable through life, character matures through the life span^48^. What is even more, studies^49^ among Syrian refugees in Sweden show that person-centered initiatives that develop Self-Directedness, Cooperativeness, and Self-Transcendence lead to decreases in mental illness (e.g., depression, anxiety) and increases in well-being (e.g., life satisfaction). Hence, character traits might represent health-related abilities that are necessary for people’s well-being and for coping with the present and future challenges of the twenty-first century^40,50,51^. The concept of the self as autonomous, responsible, resourceful, and self-acceptant (i.e., high Self-Directedness); the concept of the self as tolerant, helpful, empathic, principle, and compassionate (i.e., Cooperativeness); along with transpersonal identification, spiritual acceptance, and contemplation (i.e., high Self-Transcendence), improve inter- and intrapersonal relations, well-being, and resilience, because these aspects influence human interactions and experiences in different planes of life (i.e., sexual, material, emotional, intellectual, and spiritual)^38,50,52^. Accordingly, individuals who report high levels in all three character traits consistently report the highest levels of well-being, healthy longevity, good objective health, optimal cardiovascular health, including healthy lifestyle as well as reduced risk for chronic diseases and better heart rate variability in 24-h recordings of heart rhythms^38,53,54^. Therefore, the measurement of personality, specially character, is a valuable method to investigate the outcomes and adherence to activities that aim to promote well-being and healthy behaviors^44,55^. Ergo, integration initiatives that promote character development, besides life satisfaction and other health-related outcomes, might indicate sustainable improvements in people’s ability to adapt and regulate their thoughts, emotions, behaviors, and social life in their host country. Moreover, character at the beginning of such initiatives might predict adherence to the activities and therefore leading to positive outcomes.

### Integration initiatives

Host countries often implement integration initiatives aiming to promote health and attenuate the social risk factors detailed earlier. Few of them, however, are studied and reported in the literature (for some examples see elsewhere^56–58^) and even fewer address character development, increases in life satisfaction, and/or activity adherence—adherence is important because a major determinant of the effect of any treatment is that the participant complies and engages in the activities provided and recommended^59–61^. One such initiative between 2016 and 2018 in Blekinge, in the South Sweden, was the community project “Health for Everyone—Sport, Culture and Integration” which offered physical activity in groups of 20–30 individuals to 467 asylum seekers once a week for a 10-week period, along with cultural orientation (i.e., one visit to the museum to learn about Blekinge’s cultural heritage), and a 20-h health orientation course. The project was carried out within the program “Society orientation for newcomers”—a 60-h course administered by the municipalities in Blekinge comprising general knowledge about how Swedish society is organized, the functions of different government authorities, and practical everyday life tasks. The municipalities’ based the project on the fact that physical activity is an effective and cost-efficient tool to improve and maintain physical and mental health and well-being, increase social interaction and community engagement, and promote a sense of being part of society^62–65^. In other words, the project aimed to improve the mental health outcomes caused by pre-migration stressors (e.g., trauma exposure) and post-migration stressors (e.g., prolonged bureaucratic processes, limited language skills, discrimination, etc.). However, as stated earlier, despite the vast evidence associating physical activity with improvements in mental health^66,67^ and overall quality of life^68,69^, there is a relatively limited number of studies on physical activity initiatives among refugees and asylum seekers^70^. In addition, the amount of physical activity in the project is much lower than the adherence recommendations by the World Health Organization—that is, a minimum of 30 min of physical activity of moderate intensity on most days of the week for at least 10 min consecutively^71^. Thus, our research team was approached to evaluate the project in 2017.

Our first study^72^ showed significant increases in participants’ cardiovascular fitness and skeletal muscle mass and decreases in visceral fat area. In short, suggesting that the 10-week period, combining social-health-cultural orientations and resistance-aerobic training, yielded small but positive results in important physiological outcomes among asylum seekers. From a community perspective, this short initiative allowed a much larger number of asylum seekers to participate with less financial and logistical constraints; and still had positive impacts on participants’ physical health. Although promising, the question of possible changes in life satisfaction and character still remains.

### The present study

To investigate health promotion beyond alleviation of mental illness, we aimed to evaluate changes in asylum seekers’ character and life satisfaction after participating in the community project “Health for Everyone—Sport, Culture, and Integration”. Since the aim of the project was to increase physical and mental health and integration among participants, we expected increases in life satisfaction and character development. In addition, as detailed in the sections above, both life satisfaction and character are indicators of good physical health and markers of long-term health improvements and healthy behaviors, including adherence to treatment. Hence, we investigated if character traits and life satisfaction at baseline predicted adherence to the intervention and the changes in physiological health reported in our first study^72^.

## Methods

### Ethical statement

The evaluation of the project “Health for Everyone—Sport, Culture, and Integration” was approved by the Swedish Ethical Review Authority (Dnr. 2017/604). The study was conducted in accordance with the ethical standards of the 1964 Helsinki declaration and further amendments. Hence, all the participants were provided with the necessary information to give their verbal and written informed consent (e.g., aims of the study, voluntary participation, etcetera).

### Participants and procedure

The project “Health for Everyone—Sport, Culture and Integration” was initiated by Ronneby Municipality in partnership with Blekinge Sports Association and financed by Region Blekinge and the County Administrative Board in Blekinge. The Blekinge Sports Association designed the activities, while recruitment and selection were managed by the five municipalities in Blekinge. Attendance was scheduled within the program “Society orientation for newcomers”, which is a 60-h course comprising general knowledge about how Swedish society is organized, the functions of different government authorities, and practical everyday life tasks. Thus, most participants had just arrived at Sweden as asylum seekers and were directed to the project as part of the services provided by the municipalities. The majority of participants spoke Arabic and originated mostly from Syria (about 80%) and some from Iraq, while a minority spoke Persian (Iran) and Somali (Somalia). Nevertheless, country of origin or how much time they had spent in Sweden was not registered within the project or the evaluation study.

The project was conducted between 2016 and 2018, while the evaluation detailed here started in 2017. Every 10 weeks during this period, groups of 20–30 asylum seekers were offered physical activity once a week for 8 weeks—the first and last weeks were reserved for the physiological testing and answering the survey aimed to evaluate the project. The physical activity consisted of a combination of aerobic and resistance training in rotating exercise stations (i.e., circuit training). During the first week, participants were also invited to join a guided visit to the Blekinge Museum to be introduced to Scandinavian history and the cultural heritage of Blekinge (i.e., cultural orientation). During these 10 weeks, in conjunction with their visit to the training and testing facilities, participants also received a once-a-week 2-h lecture on health promotion with the help of professional Arabic, Persian, and Somali interpreters (i.e., a 20-h health orientation).

In the first week, participants received information about the activities with the help of interpreters and were subsequently invited to take part in the study carried out by our research group. A research assistant provided a verbal explanation (in Swedish) with the help of interpreters, regarding the project’s evaluation and data collection procedure. All information was given both verbally and in writing through an informed consent letter and those choosing to participate in the study were asked to sign the consent letter. While Arabic and Persian translations were available for all surveys, participants who spoke Somali or any other language were assisted by the interpreters who translated each question verbally from Swedish or English versions of the instruments to the relevant language. The data collection, both T1 (baseline: first week of the 10-week project) and T2 (endpoint: last week of the 10-week project), lasted for approximately 1.5 h. To increase participation for the evaluation of the project, participants were offered cinema tickets after completing the physiological tests and evaluation surveys at T1 and T2.

In total, 467 participants (263 males and 204 females) with a mean age of 35.9 years (SD = 11.9) enrolled in the project^72^. For the present study, 269 participants (i.e., nonresponse rate = 42.24%) provided background data (i.e., age and gender) and self-reported character and life satisfaction at T1 and T2. This sub-sample consisted of 151 (56.13%) males (*M*_*age*_ = 38.95 ± 10.51) and 118 (43.87%) females (*M*_*age*_ = 42.55 ± 9.87). As described elsewhere^72^, physiological health data was available for all participants in this sub-sample. See Table 1 for a detailed description of the different activities that each group underwent during the 10-week project described here.

**Table 1:** Description of the different activities that each group of asylum seekers underwent during the 10-week project “health for everyone—sport, culture and integration”.

	Timeline	Evaluation	Health for everyone—sport, culture, and integration			
			Physiological testing	Physical activity	Health orientation	Cultural orientation
Society orientation for newcomers	Week 1 T1	Life satisfaction and character	Cardiorespiratory fitness, skeletal muscle mass, body fat mass, and visceral fat		2-hour lecture on health promotion	Visit to the Blekinge museum
	Week 2			1 hour of circuit training	2-hour lecture on health promotion	
	Week 3			1 hour of circuit training	2-hour lecture on health promotion	
	Week 4			1 hour of circuit training	2-hour lecture on health promotion	
	Week 5			1 hour of circuit training	2-hour lecture on health promotion	
	Week 6			1 hour of circuit training	2-hour lecture on health promotion	
	Week 7			1 hour of circuit training	2-hour lecture on health promotion	
	Week 8			1 hour of circuit training	2-hour lecture on health promotion	
	Week 9			1 hour of circuit Training	2-hour lecture on health promotion	
	Week 10 T2	Life satisfaction and character	Cardiorespiratory fitness, skeletal muscle mass, body fat mass, and visceral fat		2-hour lecture on health promotion	

The project was scheduled within the program “society orientation for newcomers”, which is a 60-h course comprising general knowledge about how Swedish society is organized, the functions of different government authorities, and practical everyday life tasks. The participants’ attendance on the project’s physical activity segment (i.e., adherence) was registered by the instructors at the beginning of each weekly training session. Week 1 marks the baseline (T1) and Week 10 the endpoint (T2) of the project.

### Measures

#### Character

We used the Short Character Inventory, which was designed by C. R. Cloninger for Time Magazine as a brief version of the Temperament and Character Inventory^39^. The Short Character Inventory is easy to administer for testing relationships among personality variables in large groups^73^. We obtained permission from C. R. Cloninger to include the inventory in the present study. The inventory contains 15 items, five for each character trait, that are all present in the original long version of the Temperament and Character Inventory, which has been validated in 30 languages, including English, Swedish, Arabic, and Persian^74–76^. Hence, in the present study, only the few participants who had Somali as their mother tongue were assisted by the professional interpreters. The instrument asks participants to rate each of the 15 statements on a 5-point Likert scale (1 = *definitely false*, 5 = *definitely true*). Examples of the statements are: “Each day I try to take another step toward my goals” (Self-Directedness), “I enjoy getting revenge on people who hurt me” (Cooperativeness, reversed item), and “Sometimes I have felt like I was part of something with no limits or boundaries in time and space” (Self-Transcendence). The mean sum of the five items measuring each character trait is the participants’ score in each character trait, thus, ranging between 1 (i.e., low for each trait) and 5 (i.e., high for each trait).

#### Life satisfaction

The Satisfaction with Life Scale^26^ assesses the cognitive component of subjective well-being and consists of 5 items (e.g., “In most ways my life is close to my ideal”, “The conditions of my life are excellent”, “I am satisfied with my life”) that require a response on a 7-point Likert scale (1 = “*strongly disagree*”, 7 = “*strongly agree*”). The mean sum of the five items is expected to measure a single factor, thus, ranging between 1 (i.e., low satisfaction with life) and 7 (i.e., high satisfaction with life). The Satisfaction with Life Scale has been validated in several languages, including English, Swedish, Arabic, and Persian^77,78^. Hence, in the present study, only the few participants who had Somali as their mother tongue were assisted by the professional interpreters.

#### Adherence

The participants’ attendance was registered by the physical activity instructors at the beginning of each weekly training session. A total of eight times was the maximum attendance rate. Hence, we simply divided the number of recorded attendances for each participant by eight, which gave us the attendance percentage that each participant had throughout the whole project. This percentage was used as an indicator of adherence to the physical activities within the project.

#### Physiological health

The multi-stage fitness test, also known as beep-test, was used to estimate participants’ cardiorespiratory fitness (i.e., VO₂ max). The following formula is used to transform the beep test results to VO₂ max: VO₂ max = 3.46 * (Level + No. of Shuttles/(Level * 0.4325 + 7.0048)) + 12.2. Participants’ body weight, skeletal muscle mass (SMM) percentage, body fat mass, and visceral fat were calculated using measures obtained through a direct segmental multi-frequency bioelectrical impedance analysis (DSM-BIA) with an InBody 720 body composition analyzer^72^. For a detailed description of each measure see elsewhere^72^. For each physiological measure we calculated the change ratio by simply subtracting each participant’s T1 measure from its respective T2 measure. Hence, a larger score indicates an increase in each specific physiological health measure.

### Statistical treatment and analysis

All the statistical analyses were conducted using IBM’s Amos v24 and SPSS v26 software. We analyzed skewness and kurtosis of participant’s scores in all variables. For the character traits, life satisfaction, and adherence scores the values were between ± 1.96, thus, we considered the data as normally distributed. For the physiological data, 8 cases with outlier values were removed to acquire normal distribution. The data had 35.5% missing values, which is larger than the 5–10% or less missing rate that is considered acceptable^79,80^. A Missing Completely at Random test showed that the missing data were completely at random (*Chi-Square* = 51.65, *df* = 52, *p* = 0.49), that is, the missing data did not depend on the observed or unobserved data. therefore, we used multiple imputation to substitute missing values, which imputed values sampled from their posterior predictive distribution of the observed data^81^. After this treatment, we conducted a paired-samples *t*-test to measure mean differences in life satisfaction at T1 and T2 and differences in character traits (i.e., Self-Directedness, Cooperativeness, and Self-Transcendence) at T1 and T2. Next, we performed two separate structural equational models (SEM): (1) to investigate if character and life satisfaction at baseline (T1) predicted attendance percentage (i.e., adherence) and (2) to investigate if character and life satisfaction at baseline (T1) predicted changes in physiological health (i.e., change ratio of physical fitness or VO_2_ max, body weight, skeletal muscle mass percentage, body fat mass percentage, and visceral fat area).

#### Reliability of the scales

We assessed the reliability of the character and life satisfaction scales by analyzing intra-class correlation coefficients (ICC) based on research suggesting that values less than 0.50 indicate poor reliability, between 0.50 and 0.75 indicate good reliability, and between 0.75 and 0.90 indicate excellent reliability^82^. We chose ICC as a measure of reliability since research suggest that short measures’ reliability is better assessed through test–retest analyses^83,84^. Indeed, when constructing longer measures researchers have more items for each scale, which alone increases internal reliability. In contrast, for short measures researchers often sacrifice internal reliability when aiming to measure the whole construct or different aspects of it (i.e., content validity). The ICC estimates for each scale in this study ranged from moderate to excellent, with ICC-values between 0.57 and 0.80. Specifically, the ICC estimate was 0.68 for Self-Directedness, indicating good reliability; 0.57 for Cooperativeness, indicating moderate reliability; 0.71 for Self-Transcendence, indicating good reliability; and 0.80 for life satisfaction, indicating excellent reliability. These estimates were based on a 95% confidence interval. Overall, the results suggest that the character and life satisfaction scales are reliable measures for assessing personality traits and well-being in this population.

## Results

### Descriptive statistics

The average of attendance percentage in the physical activity sessions for males was 74.39% and for females 70.86%. Table 2 shows the mean and standard deviation for the character traits and life satisfaction of the whole sample and for both males and females at T1 and T2.

**Table 2:** Mean and standard deviation (SD) for character traits and life satisfaction at baseline (T1) and endpoint (T2) of the 10-week project.

Variables	Gender				Total	
	Male		Female		Mean	SD
	Mean	SD	Mean	SD		
Self-directedness T1	3.67	0.64	3.70	0.58	3.68	0.62
Self-directedness T2	3.69	0.61	3.71	0.49	3.72	0.56
Cooperativeness T1	3.79	0.56	3.79	0.49	3.79	0.53
Cooperativeness T2	3.70	0.53	3.78	0.48	3.73	0.51
Self-transcendence T1	3.83	0.72	3.95	0.64	3.88	0.69
Self-transcendence T2	3.78	0.76	3.82	0.66	3.80	0.72
Life satisfaction T1	4.49	1.27	4.77	1.26	4.61	1.27
Life satisfaction T2	4.47	1.27	4.70	1.31	4.57	1.29

### Changes in character traits and life satisfaction

A paired-samples *t*-test was performed to compare the mean scores of Self-Directedness, Cooperativeness, Self-Transcendence, and life satisfaction at T1 with their respective mean scores at T2. The results showed that only Self-Transcendence was significantly different across time points. More specifically, participants reported lower Self-Transcendence at T2 (Table 3). The effect size, however, was small (Cohen’s d = -0.11).

**Table 3:** Paired-samples t-test of the character traits and life satisfaction at baseline (T1) and endpoint (T2) of the 10-week project.

Dependent variables	Mean differences	Standard error	*t*-value	df	*p*-value	*Cohen’s d*
Self-directedness (T1–T2)	− 0.04	0.04	− 1.10	268	0.27	0.07
Cooperativeness (T1–T2)	0.06	0.03	1.61	268	0.11	− 0.12
Self-transcendence (T1–T2)	0.08	0.04	2.04	268	0.04	− 0.11
Life satisfaction (T1–T2)	0.04	0.06	0.67	268	0.50	− 0.03

### Character traits and life satisfaction at baseline (T1) as predictors of adherence at endpoint (T2) of the 10-week project

**Fig. 1:**
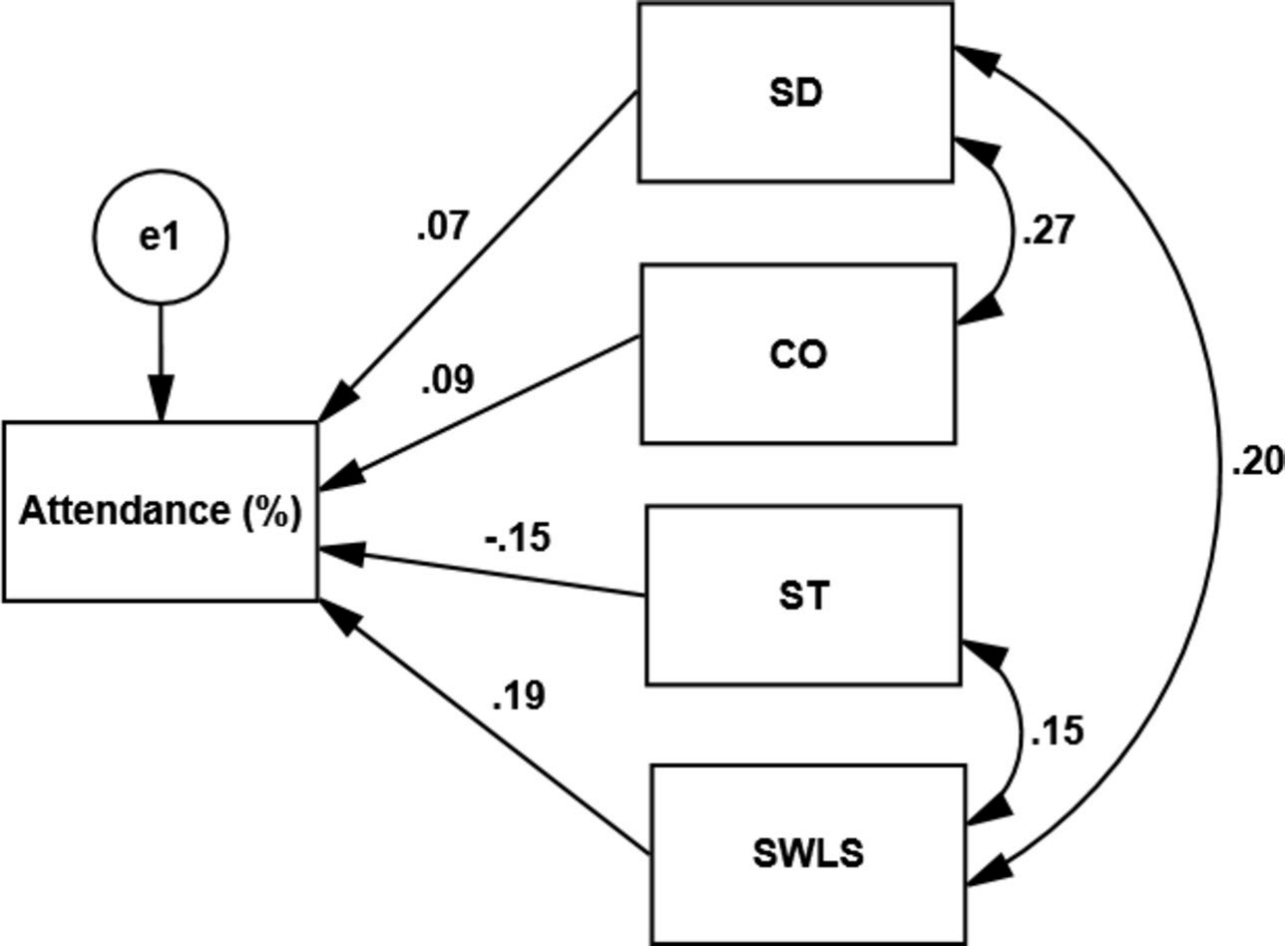
SEM model for the relationship between character traits and life satisfaction at baseline (T1) and attendance percentage (adherence) by the endpoint (T2) of the 10-week project. *SD* self-directedness, *CO* cooperativeness, *ST* self-transcendence, *SWLS* satisfaction with life. Only self-transcendence and life satisfaction predicted adherence significantly (*p* < 0.05).

The first SEM aimed to investigate if the character traits and life satisfaction at T1 predicted adherence (i.e., attendance percentage) to the physical activity sessions at T2. The analysis showed that life satisfaction and the character dimension of Self-Transcendence had a significant effect on participants’ attendance percentage to the physical activity sessions (*χ*^2^/df = 1.56, CFI = 0.97, RMSEA = 0.05). Figure 1 depicts the regression weights between character traits and life satisfaction at T1 and attendance percentage by the end of the 10-week project. Self-Transcendence had a negative or decreasing effect on adherence (i.e., attendance percentage), while life satisfaction had a positive or increasing effect on adherence (*p* < 0.05). The effect sizes, however, were small.

### Character traits and life satisfaction at baseline (T1) as predictors of changes in physiological health at endpoint (T2) of the 10-week project

**Fig. 2:**
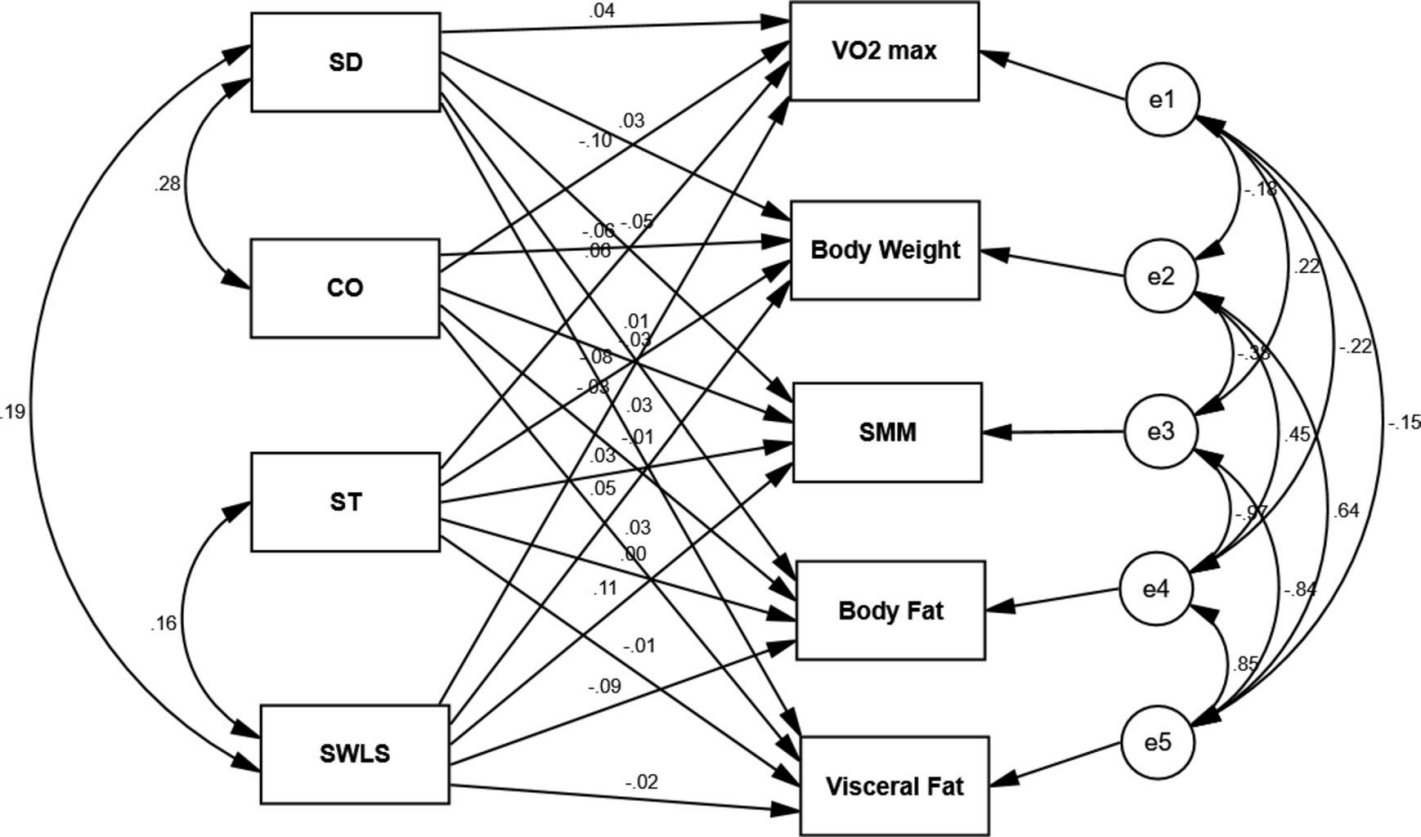
SEM model for the relationship between character traits and life satisfaction at baseline (T1) and changes in physiological health measures at endpoint (T2) of the 10-week project. *SD* self-directedness, *CO* cooperativeness, *ST* self-transcendence, *SWLS* satisfaction with life, *SMM* skeletal muscle mass. There were no significant correlations between variables.

The second SEM showed that none of the character traits nor life satisfaction predicted changes in physiological health (*χ*^2^/df = 1.40, CFI = 0.99, RMSEA = 0.04). Figure 2 displays the regression weights between character traits and life satisfaction at baseline (T1) and changes in the physiological health measures at endpoint (T2) of the 10-week project.

## Discussion

The aim of the present study was to evaluate the “Health for Everyone—Sport, Culture, and Integration” community project by investigating changes in asylum seekers’ character traits and life satisfaction and if these variables at baseline could predict adherence to physical activity and changes in physiological health. In short, the results showed that by the end of the 10-week project, participants’ Self-Directedness, Cooperativeness, and Life Satisfaction did not increase as expected. Conversely, Self-Transcendence decreased significantly at the end of the project. Moreover, high levels of life satisfaction and low levels of Self-Transcendence at baseline predicted high levels of adherence to the physical activity sessions. Put in another way, feeling satisfied with the conditions in one’s life (i.e., high life satisfaction) and having a pragmatic outlook on the world around (i.e., low Self-Transcendence) at the beginning of the project predicted higher attendance to the training activities. Finally, neither character traits nor life satisfaction predicted changes in physiological health.

We expected positive changes in character and subjective well-being since other studies show that frequent and regular participation in physical activity improve psychological health and well-being, reduce stress, and attenuate symptoms of depression and anxiety^85^. For example, it has been reported that individuals who frequently engage in physical activity experience fewer days of poor mental health when compared to sedentary individuals (i.e., individual who spend six or more hours per day sitting or lying down and lack significant physical movement on a daily basis)—mental health improvement being associated with exercise sessions of 45 min/three to five times per week^86^ and for periods of six months or longer^87^. Even low-intensity aerobic programs (i.e., 30–35 min/3–5 days per week for a period of 10–12 weeks) and moderate-intensity physical activity (i.e., a minimum of 150 min/week) are efficient in increasing positive emotions and well-being^88^. Hence, suggesting that the current recommendations^89^ and the amount of physical activity in the initiative evaluated here, are effective for physical health but not for promoting character development or well-being^90^. Indeed, the total amount of physical activity offered to the participants throughout the 10-week project “Health for Everyone—Sport, Culture, and Integration” was probably insufficient to generate relevant positive changes in character traits and life satisfaction^88^. Comparing the results of the present study with those in our previous evaluation of the same main population^72^, it is plausible to suggest that physiological health can be improved with this low amount of physical activity, but that longer and more frequent and intensive activities are needed to generate changes in people’s self-concept or character and their subjective well-being^87^. However, previous studies have shown that the effects of physical activity on subjective well-being are not necessarily linked to the amount, frequency and intensity of physical activity^91,92^.

In this context, as designed by the Municipalities, the intervention was intended to mitigate the mental health problems caused by pre-migration stressors and post-migration stressors. Thus, one possible reason for the nonsignificant findings is that physical activity might not have an effect on the life satisfaction and self-concept of someone who arrives in the country severely traumatized and additionally affected by new stressors in the host country. Here we did not have any records of how traumatized participants were but is plausible that individual differences in trauma might be behind the nonsignificant results. That being said, since life satisfaction is more related to a large extent to current stress (e.g., post-migration stressor in the current study) and physical health to traumatic stress (e.g., pre-migration stressor in the current study)^93^, it is reasonable to conclude that the intervention might have targeted the traumatic stress experienced pre-migration rather than current stress experienced post-migration—after all, our past study showed improvements in physical health^72^.

The significant decrease, albeit small, in Self-Transcendence among the refugees in the present study is remarkable. Self-Transcendence is the spiritual and existential part of our self-concept and refers to the identification of the self with something beyond the individual, thus, it can be described as spiritual acceptance, identification and union with humanity, nature, and the universe and its source^39^. As a matter of fact, self-transcendent experiences in nature, spiritual contemplation, and being moved by art, allows the person to experience awe, which improves mental and physical health through shifts in neurophysiology, lesser focus on the self, increased prosocial behavior, greater social integration, and increases in sense of meaning^94^. Is this specific connection to health and well-being that probably makes spirituality of special interest for public health^95^. Even though the “Health for Everyone—Sport, Culture, and Integration” project had a focus on physical activity, and it had socio-cultural elements; it was not effective for character development and even decreased Self-Transcendence. Together with past studies^49^, these findings suggest that character development might increase health but that increases in physical health do not develop people’s character per se. The question is though, why did Self-Transcendence decrease after the project?

Decreases in Self-Transcendence indicate a shift towards a more materialistic and fact-based worldview and moral pragmatism^39,50,96^, which are features of the contemporary Swedish society^97^. Indeed, in the World Value Survey^98^, Sweden is at the top of both secular-rational values (i.e., emotionally stable, pragmatic, liberal) and self-expressive values (i.e., pro-social, open, idealistic). Additionally, like other Scandinavian countries, Sweden is a bit of an exception regarding the normal life-span development of self-transcendent values, which in other parts of the world increase with age and are imbedded within a self-expressive society—in Sweden, however, spirituality is somewhat frown upon^99^ and decreases through the life span^48^. Other studies suggest that Swedish university students of different professions become less transcendent at the end of their educational training^100^. Hence, decreases in Self-Transcendence might be an indicator of some degree of integration and adaptation to the Swedish society^101^.

For instance, a cross-cultural comparison found that Iranians living in Iran scored higher in Self-Transcendence than native Swedes and Iranian refugees living in Sweden^101^. Moreover, the differences in Self-Transcendence were higher between Iranians living in Iran and Swedes than between Iranian refugees living in Sweden and Swedes^101^. Hence, suggesting an ongoing adaptation process occurring within the refugee population in terms of higher similarity to the Swedish population’s low Self-Transcendence. Although social integration of newcomers into the social structure of their host society^102^ is a desired outcome for this and other health and integration initiatives; decreased Self-Transcendence is certainly associated with lower levels of mental and physical health per se^38,38,39,50,94,96^. Understanding decreases in Self-Transcendence as an indicator of conforming to the host country and it being related to poor health at the same time, also gives an explanation to our results showing that increased adherence to the physical activities was associated to low Self-Transcendence at baseline and that character at baseline, in turn, could not predict positive changes in physiological health. That being said, pragmatism might lead to compliance to recommendations in a new society (e.g., adherence to an integration project), since individuals who are low in Self-Transcendence do not hold onto universal ideals, but rather adapt their views to what is practical in specific situations.

Life satisfaction at baseline on the other hand, had a positive effect on attendance percentages at the end of the 10-week project, a result that is consistent with previous studies showing that more physically active individuals across different ages and walks of life constantly report higher life satisfaction and vice versa^103–105^. Thus, our results suggest that individuals with high life satisfaction at the start of the project were already more physically active and/or more inclined to adhere to the physical activity segment of the project when compared to their less satisfied peers. Nevertheless, life satisfaction at baseline did not predict changes in any of the physiological health measures. Which, as explained earlier, has probably rather to do with the total amount of physical activity offered to the participants throughout the 10-week project than to an actual lack of relationship between life satisfaction and improvements in physical health^88^.

### Limitations, strengths, and future research

The main limitation of the present study is the absence of a control group that could have functioned as a standard for comparison to measure the actual effect of the activities on changes in character traits and life satisfaction. Indeed, this study was only an evaluation of a community project with pre- and post-measures and did not involve the design nor the assignment of participants to the activities within the initiative. Hence, randomized experimental studies are necessary in this endeavor. Another limitation is the low number of physical activity sessions offered throughout the project. Due to financial and logistical restrictions, the participants underwent approximately one hour of physical activity per week, which can be considered suboptimal. Yet another point to be considered in this evaluation is that expressions of character and life satisfaction can be significantly influenced by cultural background, which might be under or overestimated by the available instruments, potentially affecting the study’s results. Nevertheless, the instruments used to measure character and life satisfaction have been validated in over 20 different cultures and in many different populations. That being said, in the present study most of the participants spoke Arabic, but there were no other relevant sociodemographic variables (e.g., country of origin) available for analyses. Future studies with asylum seekers that speak different languages and have access to sociodemographic variables need to address the scales’ measurement invariance to test mean difference comparability between languages or countries of origin.

What our study did provide was that the scales used here (i.e., the Satisfaction with Life Scale and the Short Character Inventory) showed good to excellent test–retest reliability. Suggesting that these scales are reliable to measure life satisfaction and character among asylum seekers with similar characteristics as the ones in the present study. However, we measured subjective well-being using only its cognitive component (i.e., life satisfaction). Researchers using highly advanced statistical methods, such as Multiple Item Response Theory, have shown that subjective well-being is a multidimensional construct expressed as a latent factor that is influenced and influences the cognitive (i.e., life satisfaction), the affective (i.e., positive affect and negative affect), and the behavioral (i.e., harmony in life) components^106,107^. Hence, to truly address how well-being changes and its role in integration initiatives, future studies need to consider all components of a general subjective well-being factor^108–115^. Likewise, personality consists of both character and temperament^39^. Since their interaction (e.g., a person’s temperament and character profile rather than single traits) gives a better understanding of what makes people intelligently adapt and be resilient in changing and challenging situations^40,50,51^, future studies on integration initiatives need to measure both dimensions of personality and use person-centered methods (see for example^49,116–119^).

Moreover, within this population past research suggest that residence status, country of origin, amount of time being an asylum seeker, and other disadvantages (e.g., unemployment, racism, low income, and poor housing) are all associated with poor mental health and lack of well-being^120^. We had not access nor measured any of these variables, thus, future studies need to address these issues as relevant variables along personality and well-being. Another limitation is the nonresponse rate (42.24%). That is, only a sub-sample of 269 individuals voluntarily chose to participate in our study, out of a total population of 467 (i.e., about 58% accepted). It is well established that high nonresponse rates may negatively impact a study, contribute to biased outcomes, and impair the precision of the results^121,122^. The evaluation did not start at the beginning of the project, which probably contributed to some participants never being invited to be part of the evaluation. Importantly, although our analyses of the missing data showed that it was missing at random (i.e., the missing data did not depend on the observed or unobserved data), the data had 35.5% missing values, which is larger than the 5–10% or less missing rate that is considered acceptable^79,80^. Furthermore, it is unclear how the other parts of the project, particularly the health promotion lecture influenced the participants’ physical activity behavior. That being said, the lectures were conducted in conjunction with the physical activity sessions, thus, their effect is probably imbedded in the results presented here.

Finally, the concept of adherence to physical activity used in this study refers only to the attendance percentage of the 8 sessions but does not include measurements such as active engagement during sessions or a follow-up measurements regarding the participants’ maintenance of physical activity levels, both important aspects to achieve improvements in physical health^123^. Therefore, future studies must assess a more comprehensive set of measurements of adherence to physical activity interventions.

## Conclusions

In light of the analyses from the same project, showing that asylum seekers’ objective physiological health improved significantly^72^, it is plausible to suggest that a 10-week project implementing low frequency and intensity physical activity may improve physical health in this population because participants probably have a sedentary life and low levels of physical health to begin with due to their asylum conditions (e.g., unemployment, low income, poor housing and social network). The promotion of well-being and character, however, might need person-centered activities that focus on the whole person (i.e., body, mind, and psyche)^124^, that is, targeting physical, psychological, social, and spiritual well-being. In other words, it is important to point out that physical activity per se may not improve the well-being of asylum-seekers, for instance some physical activity programs can also be harmful to participants^91^. The promoting of well-being and character development might instead require person-centered initiatives focusing on the whole person and not only on physical activity in order to fit programs to the needs and life situation of this population. Moreover, although past research shows that character development is associated to increases in physical activity and healthy behavior, the mechanism does not seem to work the other way around; that is, increases in physical activity do no develop character. Importantly, if integration initiatives in Sweden lead to decreased Self-Transcendence, such “assimilation” might be detrimental to asylum seekers’ physical, mental, and social health in the long run.

Although this evaluation did not present significant positive changes in character or life satisfaction, this project has the potential to be used as a basis for health and integration initiatives that implement and incorporate more frequent physical activity and person-centered approaches targeting personality development to achieve long-term changes on asylum seekers’ lifestyle, well-being, and character^49^. Such interventions might benefit asylum seekers on a larger scale by providing support during their integration process into a new society, while promoting self-acceptance, social tolerance, and meaning in life through character development, especially high self-transcendence. Furthermore, person-centered interventions can have a preventive effect on mental disorders since character is one of the major single determinants of mental health and well-being^40,50,51^. This is particularly critical in the aftermath of the COVID-19 pandemic, which disproportionately affected individuals in vulnerable and marginalized populations, such as, asylum seekers^125,126^.

At the end, human beings are complex adaptive systems with a mixture of physical, psychological, social, and spiritual biopsychosocial structures. Any successful health promotion initiative should nurture all aspects of what constitutes the being, allowing the individual to engage on their own terms, and express themselves in a way they feel comfortable and safe with. Even though it is only recently that the research community has started to investigate this notion, it is neither new nor revolutionary. In his poem Satire X (written ca. 120 CE), the Roman writer Juvenal spoke of his ideals of embracing the physical and mental aspects of the individual by praying that the mind be sound in a sound body. Words penned ages ago that still echo today “Orandum est ut sit mens sana in corpore sano”.

## Data Availability

The data supporting the findings of this study are available from the research group, but restrictions apply to the availability of these data, and the data are not publicly available. In case data is requested, please contact Danilo Garcia.
